# Non-Newtonian Effects of Second-Order Fluids on the Hydrodynamic Lubrication of Inclined Slider Bearings

**DOI:** 10.1155/2014/787304

**Published:** 2014-10-22

**Authors:** Siddangouda Apparao, Trimbak Vaijanath Biradar, Neminath Bhujappa Naduvinamani

**Affiliations:** ^1^Department of Mathematics, Appa Institute of Engineering & Technology, Gulbarga 585103, India; ^2^Department of Mathematics, Sharnbasveshwar College of Science, Gulbarga 585 103, India; ^3^Department of Mathematics, Gulbarga University, Gulbarga 585 106, India

## Abstract

Theoretical study of non-Newtonian effects of second-order fluids on the performance characteristics of inclined slider bearings is presented. An approximate method is used for the solution of the highly nonlinear momentum equations for the second-order fluids. The closed form expressions for the fluid film pressure, load carrying capacity, frictional force, coefficient of friction, and centre of pressure are obtained. The non-Newtonian second order fluid model increases the film pressure, load carrying capacity, and frictional force whereas the center of pressure slightly shifts towards exit region. Further, the frictional coefficient decreases with an increase in the bearing velocity as expected for an ideal fluid.

## 1. Introduction

The study of non-Newtonian fluids has attracted the attention of several researchers, partly because of their practical applications in engineering and industry, like fluid film lubrication, the analysis of the polymers in a chemical engineering, and so forth, and partly because of the academic interest in the study of various fluid flow problems. The Newtonian fluid constitutive approximation is not a satisfactory engineering approach to most of the lubrication problems, because the modern lubricants are non-Newtonian in their character. Hence, numerous constitutive models have been proposed to predict the non-Newtonian character of the lubricants. The most commonly used lubricants are polymer solutions which are non-Newtonian in their character. Various models are developed to account for the non-Newtonian behavior of the fluid flow. In the class of non-Newtonian fluids, the second-order fluids have distinct features such as the normal stress effects in addition to possessing a large viscosity.

The lubrication characteristics of slider bearings with Newtonian fluids are presented by several investigators [1, 2]. Maday [3] used the bounded variable methods of the calculus of variations to determine the optimum load capacity of hydrodynamic one-dimensional gas slider bearings. The infinitely long slider bearings lubricated with couple stress fluids are studied by Ramanaiah and Sarkar [4] and they found that the load capacity and the frictional force increase for the couple stress fluids whereas coefficient of friction decreases. A non-Newtonian effect on the static characteristics of one-dimensional slider bearings in the internal flow regime is presented by Hashimoto [5]. He derived modified Reynolds equation for one-dimensional slider bearings and the resulting equation is solved analytically by using the perturbation technique. The non-Newtonian effects of powder-lubricant slurries in hydrostatic and squeeze film bearings are studied by Wu and Dareing [6]. They showed that the damping factor was increased with the addition of powdered graphite into the carrier fluid. The lubrication of slider bearings with a special third-grade fluid was considered by Yurusoy and Pakdemirli [7]. They used a perturbation method to obtain approximately velocity and pressure fields in the bearings and they showed that the combined effect of nonlinearity factor and beating flexibility affected the performance characteristics of slot-entry journal bearing significantly. A slider bearing with second- and third-grade fluids as lubricant was analyzed by Yürüsoy [8]. He presented the pressure distribution in the bearing analytically. The effect of second-order fluids on the performance of Rayleigh-step bearings is studied by Bujurke et al. [9]. The exponential slider bearings were lubricated with second-order fluids by Bujurke [10]. Non-Newtonian lubrication with second-order fluid is studied by Sawyer and Tichy [11]. Li et al. [12] presented the study on hydrodynamic lubrication with second-order fluid (II). These studies predicted advantageous lubrication characteristics with the second-order fluids over the Newtonian lubricants.

So far, no attempt has been made to study the lubrication characteristics of inclined slider bearings lubricated with second-order fluids. In this paper, an attempt has been made to study the performance characteristics of inclined slider bearings lubricated with second-order fluids by considering the constitutive equations proposed by Coleman and Noll [13]. This constitutive model accounts for the normal stress effects in the fluid film region. An approximate method is used to solve the highly nonlinear momentum equations, and the bearing characteristics are compared with the corresponding Newtonian case.

## 2. Basic Equations

The constitutive equation for incompressible homogeneous fluids of the second-order, based on the postulate of a gradually fading memory, was proposed by Coleman and Noll [13] and is given by

(1) $$\begin{array}{c} S_{ij}=-p\delta_{ij}+\varphi_{0}A{\left(1\right)}_{ij}+\varphi_{1}A{\left(2\right)}_{ij}+\varphi_{2}A{\left(1\right)}_{ik}A{\left(1\right)}_{kj}, \end{array}$$

where *S*_*ij*_ is the stress tensor, *p* is the pressure and *φ*_0_, *φ*_1_, and  *φ*_2_ are material constants. Tensors *A*(1)_*ij*_ and *A*(2)_*ij*_ are given by

(2) A(1)ij=vi,j+vj,i,A(2)ij=ai,j+aj,i+2vm,ivm,j,

where *v*_*i*_ and *a*_*i*_ are the components of velocity and acceleration and

(3) $$\begin{array}{c} a_{i}=\frac{dv_{i}}{dt}+v_{j}v_{i,j}. \end{array}$$

From (1), the Cartesian components of the stress tensor in the two-dimensional case are

(4) Sxx=−p+2φ0∂u∂x +φ1[2∂u∂x(u∂u∂x+v∂u∂y)+2{(∂u∂x)2+(∂v∂x)2}] +φ2[4(∂u∂x)2+(∂u∂x+∂v∂x)2],Sxy=φ0(∂u∂y+∂v∂x) +φ1[∂∂y(u∂u∂x+v∂u∂y)+∂∂x(u∂v∂x+v∂v∂y)   m+2(∂u∂x∂u∂y+∂v∂x∂v∂y)] +2φ2[(∂u∂x+∂v∂y)(∂u∂y+∂v∂x)],Syy=−p+2φ0∂u∂y +2φ1[∂∂y(u∂u∂x+v∂v∂y)+(∂u∂x)2+(∂v∂y)2] +φ1[4(∂v∂y)2+(∂u∂y+∂v∂x)].

## 3. Mathematical Formulation of the Problem

**Figure 1:**
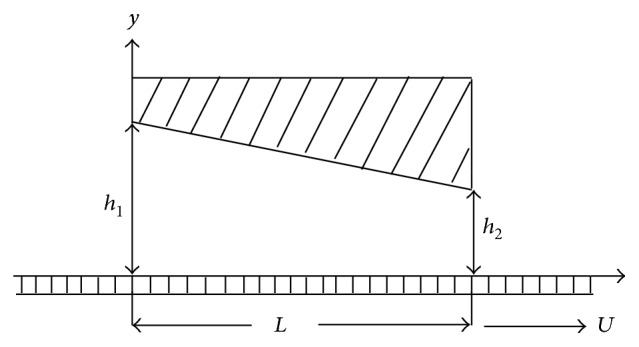
Inclined slider bearing.

We consider a bearing in which one of the two mutually opposed surfaces has a pure tangential sliding motion relative to the other surface. The geometry of the two-dimensional infinite inclined slider bearing is shown in Figure 1.

The film thickness is given by

(5) $$\begin{array}{c} H=h_{2}+\left(h_{2}-h_{1}\right)\left(\frac{x-L}{L}\right), \end{array}$$

where *L* is the length of the bearing.

The momentum and continuity equations are

(6) $$\begin{array}{c} \frac{\partial }{\partial x}\left(S_{xx}\right)+\frac{\partial }{\partial y}\left(S_{xy}\right)=0, \end{array}$$

(7) $$\begin{array}{c} \frac{\partial }{\partial x}\left(S_{xy}\right)+\frac{\partial }{\partial y}\left(S_{yy}\right)=0, \end{array}$$

(8) $$\begin{array}{c} \frac{\partial u}{\partial x}+\frac{\partial u}{\partial y}=0. \end{array}$$

Considering the nondimensional quantities:

(9) $$\begin{array}{c} \varepsilon =\frac{h_{2}}{L},\;\;u^{*}=\frac{u}{U},\;\;v^{*}=\frac{v}{V},\;\;x^{*}=\frac{x}{L},\\ y^{*}=\frac{y}{\varepsilon L},\;\;p^{*}=\frac{\varepsilon^{2}L}{\phi_{0}V},\;\;H^{*}=\frac{H}{h_{2}}, \end{array}$$

where *V* is the characteristic velocity. Since the minimum thickness of the fluid film is very small compared with the length *L*, the nondimensional parameter *ε* is a very small quantity. Substituting (9) into (6) and (7), collecting the dominating terms, and using lubrication approximation, we obtain

(10) ∂p∂x=φ0∂2u∂y2 +φ1[∂2u∂y2∂u∂x+4∂u∂y∂2u∂x∂y+u∂3u∂x∂y2   ll+∂2v∂y2∂u∂y+v∂3u∂y3]+2φ2∂2u∂x∂y∂u∂y,∂p∂y=2(2ϕ1+ϕ2)∂u∂y∂2u∂y2.        

The Boundary conditions for velocity and Pressure are

(11a) $$\begin{array}{c} u=U,\;v=0,\;\text{at}\;y=0, \end{array}$$

(11b) $$\begin{array}{c} u=0,\;v=0,\;\text{at}\;y=H, \end{array}$$

(11c) $$\begin{array}{c} \overset{H}{\underset{0}{\int }}\left(p-\tau_{xx}\right)dy=0\;\text{at}\;x=0,\;x=L. \end{array}$$

## 4. Solution of the Problem

The solution of (8) and (10) is attempted in the form

(12) $$\begin{array}{c} u\left(x,y\right)=m\left(x\right)y^{2}+n\left(x\right)y+u_{0}, \end{array}$$

where *m*(*x*) and *n*(*x*) are functions of *x* to be determined. Using (8) and the boundary conditions (11a) and (11b), we get

(13) u(x,y)=m(x)y2+n(x)y+U,v(x,y)=−dmdxy33−dndxy22,

where

(14) m(x)=3UH+CH3,n(x)=−4UH+CH2.

In the above, *C* is arbitrary constant to be determined.

Substituting (13) and (14) into (10), we get

(15) ∂p∂x=2φ0m+4(2φ1+φ2)y2d(m2)dx+4(2φ1+φ2)yd(mn)dx+(3φ1+2φ22)d(n2)dx+2Uφ1d(m)dx,∂p∂y=4(2ϕ1+ϕ2)(2m2y+mn).

Integration of (15) gives

(16) p(x,y)=2ϕ0∫m dx+4(2ϕ1+ϕ2)m2y2 +4(2ϕ1+ϕ2)mn y+(3ϕ1+2ϕ22)n2 +2Umϕ1+d,

where *d* is a constant to be determined using boundary conditions (11c).

The average pressure distribution *p* across the film thickness is

(17) p=1H∫0Hpdy,p=2ϕ0∫m dx+43(2ϕ1+ϕ2)m2H2+2(2ϕ1+ϕ2)mn H+(3ϕ1+2ϕ22)n2+d,

and the average total stress is

(18) $$\begin{array}{c} \frac{1}{H}\overset{H}{\underset{0}{\int }}\left(p-\tau_{xx}\right)dy=0\;\text{at}\;x=0,\;x=L, \end{array}$$

where

(19) τxx=1H∫0Hτxx dy=ϕ2[43m2H2+2mnH+n2].

Using (19) in (18), we get

(20) d=ϕ0L(h2−h1)(6UH+CH2)−43(2ϕ1)H2(3UH+CH3)2 +2(2ϕ1)(3UH+CH3)(−4UH+CH2) −32(ϕ1)(−4UH+CH2)2−2ϕ1U(3UH+CH3),

where

(21) C=−3U h2 d1∗,d1∗=A−A−4EDD,A=−2K[1H∗3−1]+[1H∗2−1h1∗](11−h1∗),E=K[1H∗2−1]−[1H∗−1h1∗](11−h1∗),D=−K[1H∗4−1],K=ϕ1Uϕ0L.

Use of (20) in (17) gives

(22) p=6φ0ULh2(h2−h1)[(1h1∗−d1∗2h1∗2)−(1H∗−d1∗2H∗2)] +φ2U2h22[12(1H∗−d1∗H∗2)2−6(1H∗−d1∗H∗2)  +φ1U2h22×(4H∗−3d1∗H∗2)+(4H∗−3d1∗H∗2)2] +φ1U2h22[24(1H∗2−d1∗H∗2)2−24(1h1∗2−d1∗h1∗2)2  +φ1U2h22−12(1H∗−d1∗H∗2)(4H∗−3d1∗H∗2)  +φ1U2h22+12(1h1∗−d1∗h1∗2)(4h1∗−3d1∗h1∗2)  +φ1U2h22+32(4H∗−3d1∗H∗2)2−32(4h1∗−3d1∗h1∗2)2].

The nondimensional Pressure *p* ^*^ is given by

(23) p∗=ph22φ0ULp∗=6(1−h1∗)[(1h1∗−d1∗2h1∗2)−(1H∗−d1∗2H∗2)]+φ2Uφ0L[12(1H∗−d1∗H∗2)2−6(1H∗−d1∗H∗2)  φ2φ0×(4H∗−3d1∗H∗2)+(4H∗−3d1∗H∗2)2]+φ1Uφ0L[24(1H∗2−d1∗H∗2)2−24(1h1∗2−d1∗h1∗2)2  φ2φ0−12(1H∗−d1∗H∗2)(4H∗−3d1∗H∗2)  φ2φ0+12(1h1∗−d1∗h1∗2)(4h1∗−3d1∗h1∗2)  φ2φ0+32(4H∗−3d1∗H∗2)2−32(4h1∗−3d1∗h1∗2)2].

The total load carried by the bearing across the width *b* is given by

(24) $$\begin{array}{c} W=b\overset{L}{\underset{0}{\int }}\left(p-\tau_{yy}\right)dx, \end{array}$$

where

(25) τyy=1H∫0H(2φ1+φ2)(∂u∂x)2dy=(2φ1+φ2)(43m2H2+2mH+n2).

Equation (24) can be evaluated to obtain

(26) W=b[∫0L((4h1∗−3d1∗h1∗2)26φ0ULh22(1−h1∗)   llll×{(1h1∗−d1∗2h1∗2)−(1H∗−d1∗2H∗2)}+φ1U2h2∗2   llll×{12(1h1∗−d1∗h1∗2)(4h1∗−3d1∗h1∗2)2   lllllllll−24(1h1∗2−d1∗h1∗2)2+32(4H∗−3d1∗H∗2)2   llllllll−32(4h1∗−3d1∗h1∗2)2−2(4H∗−3d1∗H∗2)2})dx].

The nondimensional load carrying capacity per unit width is

(27) W∗=Wh22φ0UL2b,W∗=∫0L(6L(1−h1∗)  ×{(1h1∗−d1∗2h1∗2)−(1H∗−d1∗2H∗2)}+φ1Uφ0L2  ×{12(1h1∗−d1∗h1∗2)(4h1∗−3d1∗h1∗2)2   l−24(1h1∗2−d1∗h1∗2)2+32(4H∗−3d1∗H∗2)2   l−32(4h1∗−3d1∗h1∗2)2−2(4H∗−3d1∗H∗2)2}dx).

The frictional force on the sliding surface of the bearing is

(28) f0=∫0L(Sxy)y=0dx,f0=φ0ULh2{L(1−h1∗)(4log⁡(1h1∗)+3d∗(1−1h1∗))   +φ1U2h2(4−3d1∗h1∗2)}.

The nondimensional frictional force becomes

(29) f0∗=f0h2φ0UL,f0∗=L(1−h1∗)(4log⁡(1h1∗)+3d∗(1−1h1∗))+φ1Uφ0L(4−3d1∗h1∗2).

In the limiting cases *φ*_1_ → 0 and *φ*_2_ → 0 the classical results of slider bearing are recovered from (27) and (29).

The nondimensional coefficient of friction *C* ^*^ can be obtained from

(30) $$\begin{array}{c} C^{*}=\frac{f_{0}^{*}}{W^{*}}, \end{array}$$

and the position of centre of pressure is

(31) $$\begin{array}{c} X=\frac{1}{W^{*}}\overset{1}{\underset{0}{\int }}x^{*}\;p^{*}\;dx^{*}. \end{array}$$

## 5. Results and Discussion

**Figure 2:**
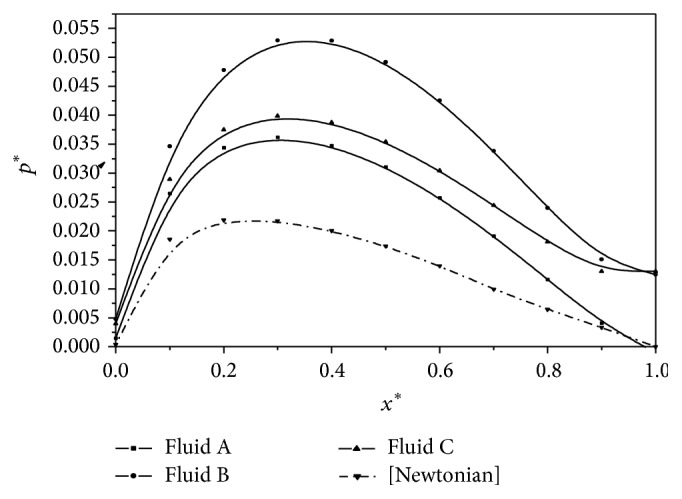
Variation of nondimensional pressure *p* ^*^ with *x* ^*^ for *U* = 0.005 m/s and *L* = 0.02 m.

**Figure 3:**
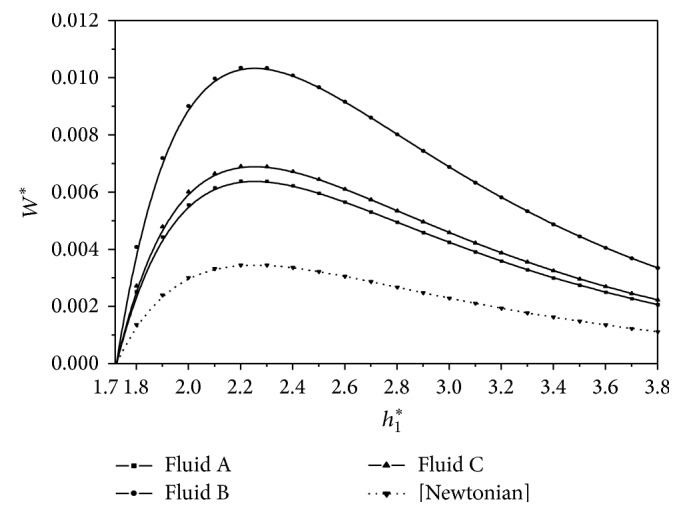
Variation of nondimensional load *W* ^*^ with film height ratio *h*_1_^*^ for *U* = 0.005 m/s and *L* = 0.02 m.

**Figure 4:**
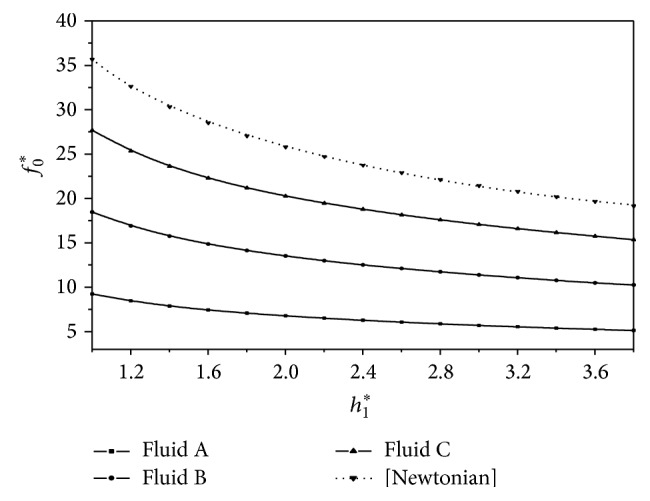
Variation of nondimensional frictional force *f*_0_^*^ with film height ratio *h*_1_^*^ for *U* = 0.005 m/s and *L* = 0.02 m.

**Figure 5:**
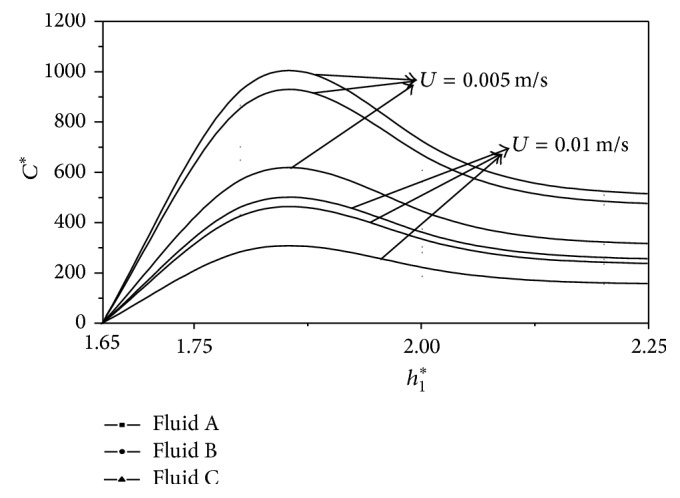
Variation of nondimensional coefficient of friction *C* ^*^ with film height ratio *h*_1_^*^ for different *U* and *L* = 0.02 m.

**Figure 6:**
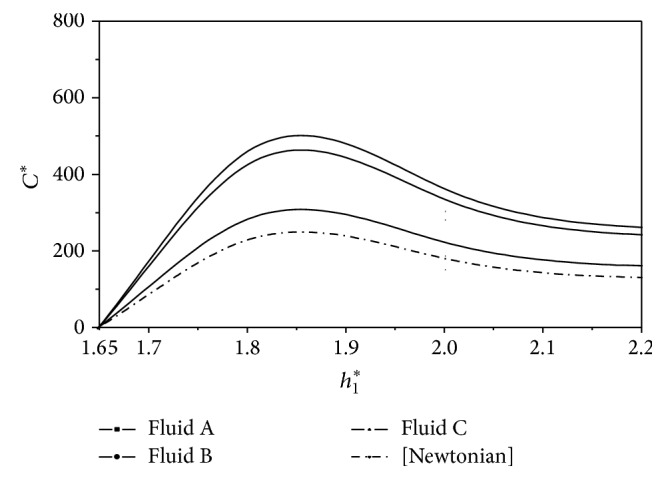
Variation of nondimensional coefficient of friction *C* ^*^ with film height ratio *h*_1_^*^ for *U* = 0.005 m/s and *L* = 0.02 m.

**Figure 7:**
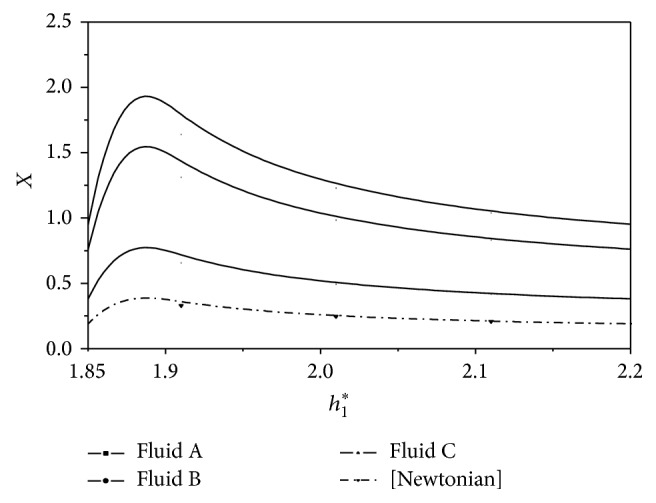
Variation of nondimensional centre of pressure *X* with film height ratio *h*_1_^*^ for *U* = 0.005 m/s and *L* = 0.02 m.

This paper predicts the non-Newtonian effects of second-order fluids on the performance characteristics of inclined slider bearings. The numerical computations of the slider bearing characteristics, namely, the nondimensional pressure *p* ^*^, the nondimensional load carrying capacity *W* ^*^, the frictional force *f*_0_^*^, the coefficient of friction *C* ^*^, and the centre of pressure *X*, are performed for various values of the material parameters *ϕ*_0_, *ϕ*_1_, and *ϕ*_2_ and the velocity *U* and the film thickness ratio *h*_1_^*^. These results are presented in Figures 2, 3, 4, 5, 6, and 7. The slider bearing performance is analyzed for three second-order fluid samples A, B, and C whose material parameter values are given in Table 1.

Figure 2 shows the variation of nondimensional pressure *p* ^*^ with *x* ^*^ for the three different second-order fluid models. The dotted curve in the graph indicates the Newtonian case. It is observed that the fluid film pressure is larger for the second-order fluids as compared to the corresponding Newtonian case. Further, it is also noted that the maximum pressure *p*_max⁡_^*^ is attained at slightly larger values of *x* ^*^ for the second-order fluids as compared to the corresponding Newtonian fluids. The variation of nondimensional load carrying capacity *W* ^*^ with the film height ratio *h*_1_^*^ is depicted in Figure 3 for the three second different order fluid samples. The results are compared with the Newtonian case (dotted curve). It is interesting to note that an increase of nearly 40% in *W* ^*^ is observed for the second-order fluid sample C as compared to the corresponding Newtonian case. The variation of nondimensional frictional force *f*_0_^*^ on the sliding surface with *h*_1_^*^ is shown in Figure 4 for different second-order fluid models. It is observed that *f*_0_^*^ decreases with *h*_1_^*^. Further, it is also observed that *f*_0_^*^ is larger for the second-order fluids as compared to the Newtonian fluids.  Figure 5 shows the variation of coefficient of friction *C* ^*^ with *h*_1_^*^ for different second-order fluid models with two values of *U*. It is interesting to note that the coefficient of friction decreases for the second-order fluids as compared to the Newtonian case. Further, it is also observed that *C* ^*^ decreases over increasing values of *U*, a desirable characteristic of lubricants [16, 17]. These results may be attributed to the significant increase in *W* ^*^ for the second-order fluids as compared to the Newtonian fluids. The variation of the centre of pressure *X* with *h*_1_^*^ is depicted in Figure 7 for second-order fluids under consideration. It is observed that the centre of pressure slightly shifts towards the entry region for the second-order fluids as compared to the Newtonian fluids.

## 6. Conclusions

The non-Newtonian effects of the second-order fluids on the lubrication characteristics of inclined slider bearings is analyzed by considering the constitutive equations proposed by Coleman and Noll [13]. The closed form expressions obtained for the slider bearing characteristics reduce to the corresponding Newtonian case expressions in the limiting cases of *ϕ*_1_ → 0 and *ϕ*_2_ → 0. According to the results presented in the above section, the following conclusions are drawn.

(1) It is observed that the fluid film pressure is larger for the second-order fluids as compared to the corresponding Newtonian case.
(2) The effect of second-order fluids is to provide significant increase in the load carrying capacity and a decrease in the coefficient of friction as compared to the Newtonian lubricants.
(3) An increase of 40% in load carrying capacity *W* ^*^ is observed for the second-order fluid sample C as compared to the Newtonian case.

These are the encouraging results for the lubrication engineers in the efficient design of slider bearings.

## Figures and Tables

**Table 1:** Material parameters for polyisobutylene in cetane at 30°C [14] and osteoarthritic fluid [15].

Fluid description	ϕ_0_ Nsm^−2^	ϕ_1_ Ns^2^m^−2^	ϕ_2_ Ns^2^m^−2^
Fluid A (osteoarthritic fluid)	2.5	−0.025	0.05
Fluid B (polyisobutylene) (5.39%)	18.5	−0.3	1.2
Fluid C (polyisobutylene) (5.4%)	18.5	−0.2	1.0

## Nomenclature

*b*: Width of the bearing
*C*: Coefficient of friction
*C*^*^: Nondimensional coefficient of friction. *C* ^*^ = *f*_0_^*^/*W* ^*^
*f*_0_: Frictional force
*f*_0_^*^: Nondimensional frictional force. *f*_0_^*^ = *f*_0_ *h*_2_/*φ*_0_ *UL*
*H*: Film thickness. *H* = *h*_2_ + (*h*_2_ − *h*_1_)((*x* − *L*)/*L*)
*h*_1_: Minimum film thickness
*h*_2_: Maximum film thickness
*h*_1_^*^: Film thickness ratio. *h*_1_^*^ = *h*_1_/*h*_2_
*L*: Length of the bearing
*p*: Pressure in the film region
*p*^*^: Nondimensional pressure. *p* ^*^ = *ph*_2_^2^/*φ*_0_ *UL*
*U*: Velocity of slider
*W*: Load carrying capacity
*W*^*^: Nondimensional load carrying capacity. *W* ^*^ = *Wh*_2_^2^/*φ*_0_ *UL* ^2^ *b*
*x*, *y*, *z*: Cartesian coordinates
*x*^*^: *x*/*L*
*X*: Centre of pressure
*φ*_0_, *φ*_1_, *φ*_2_: Material constants.
